# Microscopic charge fluctuations cause minimal contrast loss in cryoEM

**DOI:** 10.1016/j.ultramic.2018.01.011

**Published:** 2018-04

**Authors:** Christopher J. Russo, Richard Henderson

**Affiliations:** MRC Laboratory of Molecular Biology, Francis Crick Avenue, Cambridge CB2 0QH UK

**Keywords:** Low-dose electron microscopy, Single-particle reconstruction, Charging, CryoEM, Structure determination,

## Abstract

The fluctuating granularity or “bee swarm” effect seen in highly defocussed transmission electron micrographs is caused by microscopic charge fluctuations in the specimen created by the illuminating beam. In the field of high-resolution single particle electron cryomicroscopy (cryoEM), there has been a concern that this fluctuating charge might cause defocus-dependent Thon ring fading which would degrade the final image. In this paper, we have analysed the 2.35 Å fringes from the (111) reflection in images of gold nanoparticles embedded in amorphous ice. We show that there is a small, yet detectable amount of defocus-dependent blurring of the lattice fringes when compared with those from a pure gold foil. The transverse electric field associated with the fluctuating charges on the insulating frozen water specimen deflects the electron beam locally and causes image blurring. The perturbation is small, decreasing the amplitude of the 2.35 Å reflection at 10 µm defocus by about 7% (intensity by 14%). For smaller defocus values in the range 2–4 µm and for resolutions that are typical in cryoEM, the effects of source incoherence and the bee swarm effect are negligible for all reasonable cryoEM imaging conditions, assuming that a field emission gun (FEG) is used for illumination. This leaves physical movement of the specimen due to radiation damage as the outstanding problem and the major source of contrast loss in cryoEM micrographs.

## Highlights

• A physical account of charge fluctuations in low-dose cryoEM is presented.
• We quantify the bee swarm charging phenomenon on cryoEM specimen.
• We measure the envelope function caused by charge fluctuations.
• The effects of these fluctuations are negligible in cryoEM.

## 1 Introduction

Specimen charging has been known to be an experimental problem for imaging non-conducting specimens since the beginning of electron microscopy. Thin films of insulating materials, such as plastic film or sections, can charge up to thousands of volts and even fragment if the potential gets high enough [1]. Introduction of an objective aperture [1] or a thin conducting film above the specimen [2] can often stabilise the charge on the specimen once an equilibrium is reached between further charging of the specimen and its neutralisation by electrons back-scattered from the objective aperture or forward-scattered from a film above the specimen. Mahl and Weitsch [3] and Dove [4] noted the presence of a characteristic “bee swarm” contrast pattern in “shadow” (i.e. highly defocussed) images of insulating specimens. Dove estimated that this was caused by fluctuating charges in the specimen, which generated transverse electric fields between 10^4^ and 10^6^ V/cm. Curtis and Ferrier [5] further developed a quantitative estimate of this random fluctuating surface charge and estimated fields of around 10^5^ V/cm. This corresponds to a change in surface potential of about 1V over a distance of 0.1 µm. Subsequent papers have reported quantitative generation of a positive surface potential from 0.3 V to 60 V that builds up during steady illumination of semiconducting thin films [6], [7], [8], and occasionally creation of negative charge in thin carbon films under special conditions [9]. There is also the so-called “Berriman effect”, named after the person who first noticed it [10], [11], [12], where brief illumination of a region of the specimen results in a patch of positive charge that appears dark in subsequent highly defocussed low magnification images. The dark patch fades rapidly as the charge is neutralised by the electron beam used to record the diagnostic images. Finally, there is the recently observed “inverse-Berriman” effect, where regions surrounding the positively charged “Berriman” patch accumulate negative charges that are also rapidly dissipated by subsequent low intensity imaging [13]. In this paper, we use images of 10 nm gold nanoparticles suspended in amorphous ice over holes in gold foils to demonstrate that there is a small amount of defocus-dependent fading of the particle lattice fringes, which is convoluted with the envelope function [14] imposed mostly by the spatial coherence of the field emission gun (FEG) of the electron microscope being used. We also show that the amorphous ice in these holes exhibits the bee swarm effect and that the magnitude of this effect is reduced on gold versus carbon foils.

## 2 Materials and methods

**Fig. 1:**
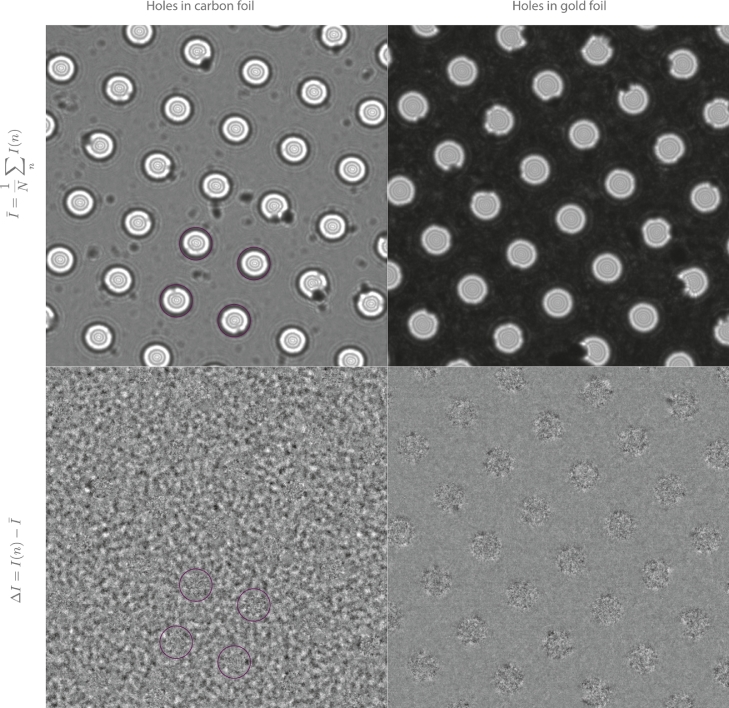
Bee swarm effect on bare carbon and bare gold foils. Top panel: raw images of bare holey carbon (left) and bare holey gold foils (right). Bottom panel: single frames of movie after subtraction of average intensity at each pixel, showing counting statistics in the holes, and the bee swarm effect on the carbon film on the bottom left panel. Circles highlight the same region on the micrographs, and the bee swarm effect is shown dynamically in Supplementary Movies 1 and 2. The fluctuations on the gold foil are much lower, partly because the beam is attenuated due to scattering, and partly due to the absence of the bee swarm effect on the electrically conductive gold foil. Each frame of the series was created using 300 keV electrons, −15 mm of defocus, $3\times 10^{-4}$ e${}^{-}/$Å^2^, 81K specimen temperature and is cropped to 10 × 10 µm.

**Fig. 2:**
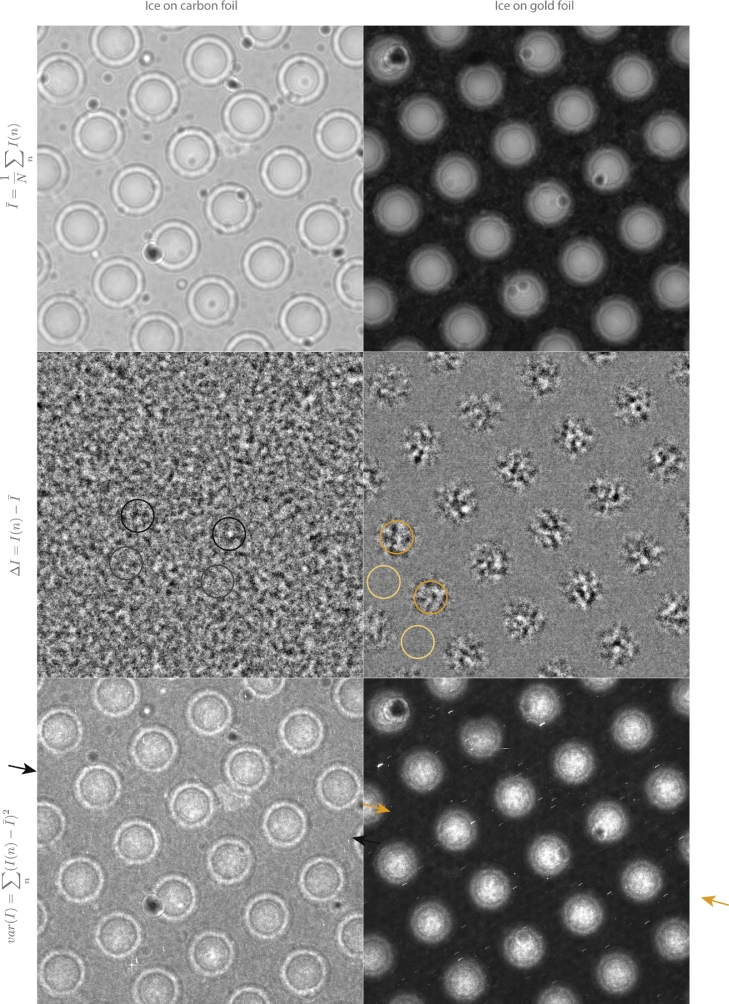
Bee swarm effect on holey carbon and holey gold with plunge-frozen amorphous ice. Top panel: average intensity in movies of ice on carbon and ice on gold foils. Middle panel: single frame from movie after subtraction of average intensity at each pixel. Bottom panel: variance over the entire sequence of movie frames. This shows that the microscopic charge fluctuation (bee swarm effect) occurs with amorphous ice specimens made by the normal Dubochet plunge-freeze method. Each frame of the series was created using 300 keV electrons, −15 mm of defocus, $4\times 10^{-4}$ e${}^{-}/$Å^2^, 81K specimen temperature and is cropped to 10 × 10 µm.

The specimen supports used to demonstrate the bee swarm effect in Fig. 1 were a carbon foil on a gold grid (Quantifoil Au 300 mesh 1.2/1.3 [15]) to show the effect, and gold foils on a gold grid (UltrAuFoil 300 mesh 1.2/1.3 [16]) as controls that do not show the large fluctuations in intensity. Images were recorded as 100 frame movies at 300 keV on an FEI Polara G2 electron microscope at a magnification of 900 ×  with an exposure of 3$\times 10^{-4}$ e${}^{-}$/Å^2^/s (1s frames) at - 15 mm of defocus. The grids and imaging conditions used to demonstrate the bee swarm effect on amorphous ice in Fig. 2 were the same as above, except with 4×10^−4^ e^-^/Å^2^/s. For the images of gold nanoparticles in ice, a specimen of 10 nm unconjugated gold colloid (BBInternational) was concentrated 20 - fold by centrifugation at 4 °C for 10 min at 13,000 rpm in an Eppendorf 514D benchtop centrifuge, and the resulting soft pellet was resuspended in pure water. Samples of 3 µL were then applied to gold grids, (UltrAuFoil R 0.6/1.0 300 mesh), blotted for 10 s and plunge-frozen using a controlled environment vitrification system [17] and a precision cryostat [18] inside a cold room at 4 °C. Grids of pure water for the experiments in Fig. 2 were prepared in the same way but with double distilled, filtered water (resistivity  > 18 MΩ cm). Images were recorded at 300 keV on an FEI Polara G2 electron microscope at a nominal magnification of 115,000 ×  using a Falcon III direct electron detector in integrating mode. The pixel size was 0.942 Å (calibrated magnification 148,600  × ) with 2 s exposures at 32 frames/s, using an electron dose of 0.3 e${}^{-}$/Å^2^/frame. Other electron optical conditions were spotsize 4, C_2_ aperture 30 µm and objective aperture 70 µm, which excludes all except the (111) diffraction from the gold nanoparticles. On each fresh 0.6 µm hole, a series of dose-fractionated images was recorded with the objective lens focus changed in 1 µm steps from nominally 1 µm overfocussed to 10 µm underfocussed. Thus, for each new hole, 11–13 movies were recorded with different defocus settings. It quickly became clear that the first image in each series (0.3 e${}^{-}$/Å^2^/frame or 19 e${}^{-}$/Å^2^ for the whole 64 frame micrograph) suffered from beam-induced motion and the next two or three also showed significant blurring. It was not until the fourth or fifth image in each series (i.e. after an electron dose of 80–100 e${}^{-}$/Å^2^) that the fringes from the gold nanoparticles could be seen in every frame without any frame-to-frame motion correction. To obtain images of gold under the same conditions without ice for comparison to those of the particles embedded in ice, we used the edges of the supporting gold foil in the following way. The residual ice was burned off from the edge of an empty hole using a high dose, and then a defocus series was recorded under identical conditions with defocus values from 1 to 10 µm underfocus.

To calculate quantitatively the image contrast and allow it to be plotted as a function of defocus, as shown in Fig. 6, the frames of each movie were first aligned using the program UNBLUR [19]. Each image was then examined to find bright diffraction beams from gold nanoparticles that were oriented in diffracting positions, as seen with the boxed particle in Fig. 4(a) that has two diffraction beams. Only those particles that were in diffracting orientations throughout the entire defocus series were selected. Typically less than one-third of the gold particles were in diffracting orientations, and perhaps only one-third of those would remain in a diffracting orientation (i.e. they did not rotate) during the whole series. Each gold particle might rotate by up to one degree during each 2 s exposure, so that the strength of the diffraction spots varied, and often appeared or disappeared entirely from one image to the next. This was expected since it is well known that the gold particles and large viruses move and rotate during the exposures [20]. Nevertheless, there were enough gold particles that remained in diffracting orientations that we were able to identify 7 sets of gold fringes from 5 gold nanoparticles in two series of images that persisted throughout the defocus series. The measurements of image contrast were made by dividing the integrated intensity of the 2.35 Å gold lattice peaks in the Fourier transforms of the area in each diffraction spot by the integrated, background-corrected density for each diffracted beam in the original image of the summed movie frames. This procedure effectively normalised the measurements of image contrast so that it was independent of the size of the gold particle and independent of the exact orientation of the particle in each image. Based on the ratio of the fringe spacing (2.35 Å) to the particle size (100 Å), we estimate that the gold particles would need to change orientation by 1.5° to change from being in a maximally diffracting position to one where the diffraction had dropped to less than 20% of maximum. The resulting plot and estimation of standard deviation was obtained by averaging the measurements from 7 diffracted beams from 5 gold nanoparticles in two defocus series.

To calculate the defocus dependence of the intensity of the 2.35 Å diffraction fringes from the edges of the gold foils, it was not possible to directly normalise the intensity in the reflections since the diffraction was less regular and often overlapped with diffraction from the multiple crystals in the thick gold foil. Instead we used a simple integrated intensity and plotted how this changed with defocus. We averaged measurements from 6 different gold crystallites from 3 different images. Fortunately, because the gold crystallites in the continuous foils were solidly anchored to the grid, they did not undergo any beam-induced reorientation of motion, so normalisation was not necessary. Finally, the fitted curves in Fig. 6 were plotted as a Gaussian centred at F=0.14 µm (representing the offset at 2.35 Å due to the Polara spherical aberration of $C_{s}=2.0$ mm) for the gold-on-gold envelope function and as a Gaussian centred at F=0.0 µm for the additional envelope function for the gold-in-ice particles, which we interpret as being due to the microscopic electrostatic charge fluctuations.

## 3 Results

### 3.1 Bee swarm effect

We first demonstrate the bee swarm effect as observed by Dove [4]. Fig. 1 shows images of bare holey carbon (QuantiFoil [15]) and bare holey gold (UltrAuFoil [16]) grids. The bottom panel shows the difference between a single frame and the average in both cases. On these grids where the electron beam passes through the hole, the granularity and noise statistics represent simple stochastic noise from the Poissonian distribution in each pixel due to the limited electron flux. On the carbon foil, which has poor conductivity, there are large fluctuations in intensity (i.e. bee swarm effect) with a distribution that has a peak spatial periodicity of the order of 100 nm. The supplementary information includes movies from both types of foil, showing how the bee swarm rapidly fluctuates on each frame from the poorly conducting carbon foil, but is not observed on gold foil.

**Fig. 3:**
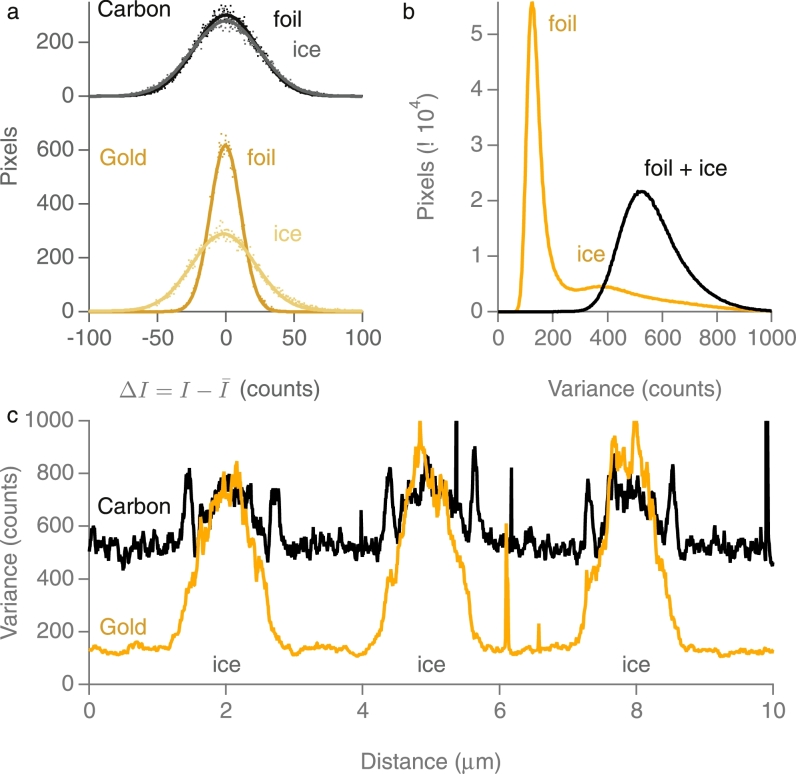
Histograms (a) of $I-\bar{I}$ at each pixel over the holes with ice and over the adjacent gold and carbon foils. The fluctuations over the ice are greater than over the foil for both gold and carbon. (b) histogram of variance over whole image showing that the bee swarm fluctuation are lower for ice in holey gold foils than for ice on carbon foils. (c) plot of variance along the lines shown between arrows in the bottom panel of Fig. 2.

We next demonstrate that these microscopic charge fluctuations also occur on typical ice-embedded specimens as used currently for single particle cryoEM. Fig. 2 shows the raw images (top) and the difference (middle) between an individual frame and the average from images of pure amorphous ice on holey carbon and on holey gold grids with similar sized holes, made using the Dubochet plunge-freeze approach [21]. Fig. 3 provides a quantitative depiction of the information in the images. The fluctuations are greatest in the ice regions in the holes, as expected since amorphous ice is an insulator [22].

**Fig. 4:**
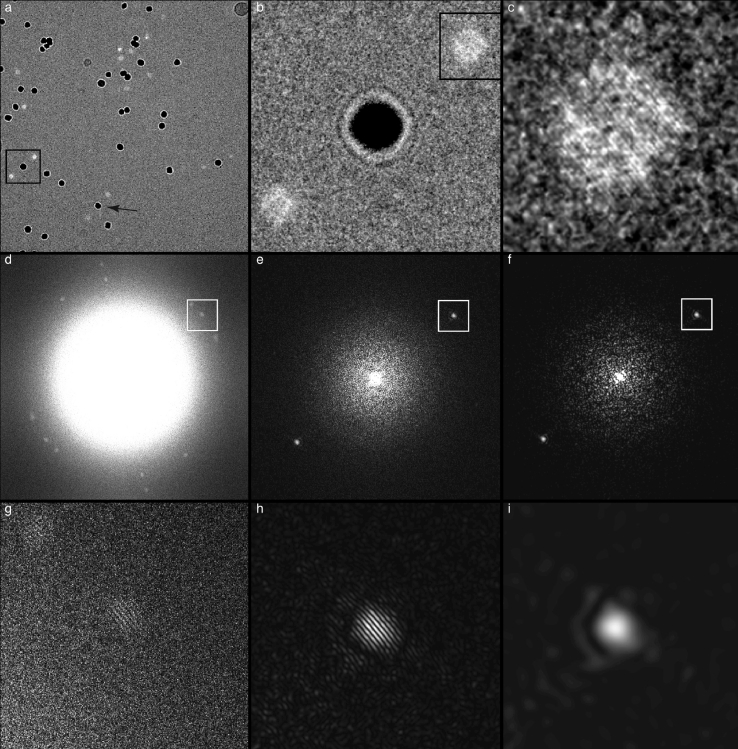
Composite panel showing images of (a) most of the area of an entire hole, (b) one gold particle (plus two diffracted beams) and (c) one diffracted beam showing gold lattice fringes. The first row shows the images in real space. The second row (d, e, f) shows the entire Fourier transform (FFT) of the areas in (a, b, c), and the third row (g, h, i) shows selected areas of the FFTs from (d, e, f). The Thon ring contrast transfer function fringes are clearly visible in (g) and (h) but not in (i). Note that the diameters of the of the diffraction spots from the gold particles, magnified in panels (g, h, i), are inversely related to the particle size (i.e. 1/10 nm${}^{-1}$) and the fringes in panels (g, h) result from the imposition of the Thon rings on the spots. The width of the areas shown in (a, b, c) are 3800, 500 and 120 Å respectively. The edge of the FFT in (d, e, f) is at 1/1.92 Å${}^{-1}$. This is image number 234757 with 3 µm defocus.

### 3.2 Imaging of gold nanoparticles in ice

**Fig. 5:**
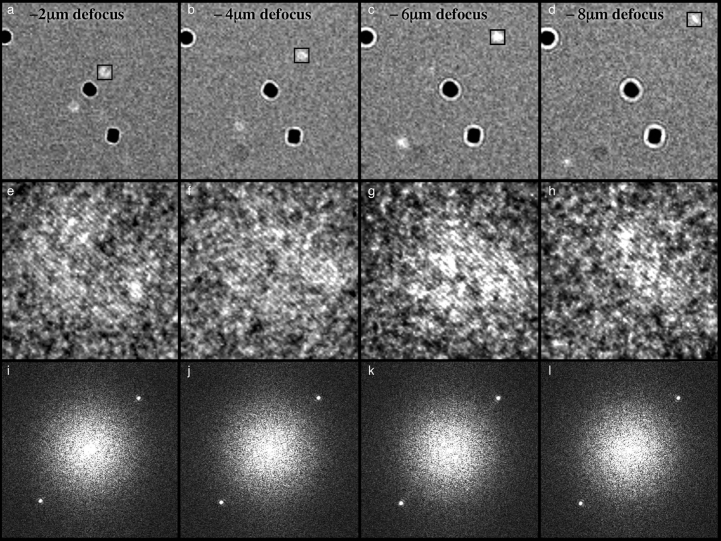
Each column shows an image of a single gold nanoparticle, a magnified view of the diffracted sideband, and the Fourier transform of the sideband image. The four columns are data collected at four different defocus values on the same particle, showing the gold fringes and similar power in their 2.35 Å diffraction spots. More careful examination shows that the central gold particle in (c) is in a more favourable orientation, so the dark-field spot is brighter in (c) and correspondingly in (g). The diffraction spots in (k) are consequently also brighter. There is a small change in magnification with defocus due to slightly divergent illumination.

**Fig. 6:**
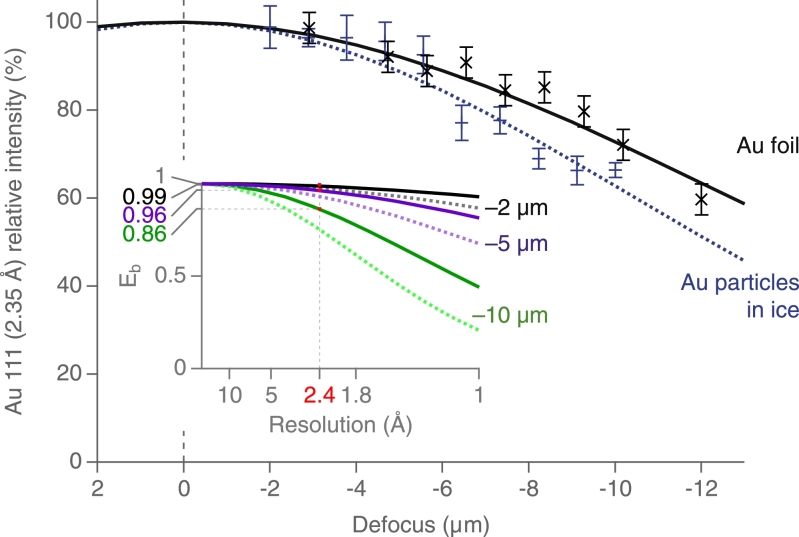
Shows how the power in the Fourier transform of images of gold fringes fades with the amount of defocus. The X symbols (and full line fit) for Au foil show measurements for a specimen with excellent electrical conductivity, namely the 2.35 Å gold fringes from (111) reflections from small gold crystals at the edge of a thin gold film. This slow fading is correlated precisely with the intrinsic high spatial coherence of the FEG source. Use of more coherent illumination allows the retention of higher contrast in the lattice fringes at higher resolution. Good images can be obtained even at 10 µm defocus. The + symbols (and dashed line fit) for the Au particles in ice show the power in the Fourier transforms of the 2.35 Å fringes in images of the (111) reflections from 10 nm gold nanoparticles that have been embedded in a thin film of amorphous ice. The slightly faster fading than observed in images of the fringes from solid gold specimens is caused by microscopic electrostatic fluctuations due to fluctuating surface charge on the insulating amorphous ice. The inset shows the envelope function (*E_b_*) that is due solely to microscopic electrostatic fluctuations, both as a function of resolution with the 2.35 Å gold spacing marked for three different defocus values at 300 keV (full lines) and extrapolated to 100 keV (dashed lines).

Fig. 4(a) shows a typical region of a thin film of amorphous ice with suspended 10 nm gold particles. The bright diffraction beams from the gold (111) reflections can be seen near each particle that is in a diffracting orientation. The same area is shown at increasing magnification (Fig. 4b–c) so that the gold lattice fringes are easily observed. The middle panels in Fig. 4 show the power spectra of the whole image and the sub-areas above. Finally, the bottom panel shows the small region around the diffraction spots in the power spectrum of the whole image and the sub-areas. The contrast transfer function (CTF) sampling of the diffraction pattern (Thon rings) is clear in Fig. 4g and h, whereas the single-sideband diffraction in Fig. 4i shows no interference pattern. There are around 300 Thon ring maxima between the origin and the 2.35 Å peak in Fig. 4g. The image that is shown in Fig. 4 was underfocussed by 3 µm, but the (111) fringes are equally well observed in overfocussed (not shown) and much more highly underfocussed images. The gold nanoparticle marked with an arrow in Fig. 4 and its (111) diffraction spots at increasing defocus values are shown in four images that make the top panels of Fig. 5. The magnified sub-areas and their diffraction patterns are shown below each of the images, which were recorded successively. There is no obvious difference between the fringe contrast or their power spectra as the amount of underfocus is increased; these and other similarly recorded images have been analysed quantitatively in the final figure. Fig. 6 shows how the power of the Fourier transform of images of 2.35 Å gold (111) fringes fades with the degree of underfocus when recorded from specimens in which the gold is in good electrical contact with and forms an intrinsic part of the supporting gold foil. The data in Fig. 6 were calculated using the motion corrected [19], summed frames to measure the strength of the FFT intensities for the gold fringes. The intensities were also measured by summing the power spectra of the individual frames [23], which gave essentially the same result. This slow fading of the envelope function demonstrates that the FEG electron source has high spatial coherence because its high brightness allows a small source size for reasonable exposure times, and that other electron-optical instabilities have also been minimised. Fig. 6 also shows the same measurement of the power in the Fourier transform of the 2.35 Å fringes from 10 nm gold nanoparticles that have been embedded in a thin film of amorphous ice, in which they are electrically isolated from the surrounding gold supports. There is a detectably greater fading of the power of the fringes for the gold particles embedded in ice compared with the fading of fringes from the gold that is attached to the gold support in the absence of ice. We found the presence or absence of an objective aperture did not affect the visibility of the fringes from the gold; all the data presented here were collected using a 70 µm objective aperture.

## 4 Discussion

The observation of only a small amount of defocus-dependent Thon ring fading from images of gold nanoparticles in ice shows that images of ice-embedded biological structures recorded far from focus should contain almost as much high resolution information as those recorded closer to focus. The coherence of the FEG sources that are now in wide use allows the retention of contrast at high resolutions even in highly defocussed images. In Fig. 6, there is a 13.5% drop in the envelope function amplitude (27% in intensity) for the 2.35 Å gold fringes at 10 µm underfocus due to the intrinsic spatial incoherence of the source and any other electron-optical contributions, whereas the additional loss of contrast for gold nanoparticles embedded in ice, which we ascribe to microscopic charge fluctuations (bee swarm effect) amounts to only 7% on amplitude (14% on intensity). Note that microscopic charge fluctuations within the gold particle will be immediately screened by conduction band electrons, but the transmitted and diffracted beams will still be affected by the unscreened charge fluctuations in the surrounding ice. The micrographs in this paper were all recorded with 2 s exposure times using a direct electron detector in integrating mode. If we had used the detector in counting mode, as is routinely done in cryoEM data collection, it would have been necessary to increase the exposure times by a factor of 20 to avoid coincidence losses in the detector. This reduction in the illumination intensity would have translated into an increase in spatial coherence, so the loss of contrast due to spatial incoherence would have been even less than observed. In addition, if we take a simple estimate as to how the effects of this charge fluctuation will scale with energy (i.e. that to first order, it scales as *v*^2^/*c*^2^ Fig. 6 inset), we find that even at 100 keV the reduction in the 2.35 Å reflection at - 5 µm defocus is only a few percent. It is thus safe to conclude that the effects of source incoherence and the bee swarm effect are negligible for all reasonable cryoEM imaging conditions. Note that any degradation in image contrast due to partial temporal coherence would affect equally all the images we have recorded independent of defocus. For modern electron microscopes, temporal coherence only becomes a factor a resolutions beyond those considered here.

The possibility of contrast loss due to charge fluctuations had been a concern discussed in recent reviews [24], [25], but fortunately we can relegate it to being a minor concern. It is now clear that beam-induced mechanical specimen motion due to radiation damage is the outstanding problem and the major source of contrast loss in cryoEM images of biological structures. The conclusions on charging reached in this paper encourage us to concentrate on these other factors in future. There has also been recent excitement from the development of the Volta phase plate (VPP) that has allowed high-resolution structures of both the proteasome [26] and haemoglobin [27] to be obtained. It was possible that a principal advantage of phase plates for biological structure determination might have been that they allowed the use of images recorded closer to focus to minimise any loss of contrast at high resolution due to microscopic charge fluctuations. Since we have shown here that there is negligible fading of Thon rings either from poor spatial coherence of the electron (FEG) source or from microscopic charge fluctuations (bee swarm effect) even at very high defocus values, we thus cast doubt on any potential advantage of imaging at decreased defocus. This also implies that an alternative approach to the use of phase plates might be to record images with higher defocus than normally used (e.g. 4 µm or greater) followed by the application a traditional CTF correction to the entire raw image before particle picking, or using the procedure we describe below and discuss in more detail in an accompanying paper [28]. Prior CTF correction of the entire image is preferable because the delocalisation of high-resolution information makes it necessary to use large box sizes for CTF correction if carried out at the single particle level. Such a procedure and the subsequent CTF correction is often called in-line holography [29]. In-focus images using a Zernike type phase plate can in principle provide up to a two-fold improvement in high resolution contrast for single particle images [30], provided that the desired *π*/2 phase shift can be maintained at all spatial frequencies. If an as yet to be constructed, “perfect” phase plate were used with an aberration corrector to collect images with zero spherical aberration that were in focus to within 50 Å across the entire field of view, this would indeed provide a two-fold improvement since the CTF zeros would be absent. Currently, practical considerations still limit the ability to accurately and repeatedly achieve the stringent conditions needed for in-focus contrast (phase shift of *π*/2, defocus and spherical aberration = 0) so that recent high-resolution structures were still determined with a small but finite defocus ( ∼ 5000 Å) [26], [27]. Further experimental work will be required to quantitatively compare the relative benefits of the two approaches, namely single-particle data acquired via in-line holography at high defocus, as described above, or phase plate data acquired with a small amount of defocus and thus some signal delocalisation and high resolution loss [26], [27].

There is a further opportunity to be realised by imaging with larger defocus. As can be seen, especially in Fig. 5, the two diffracted beams are recorded on opposite sides of the image. This means they sample different points in reciprocal space, because of curvature of the Ewald sphere. By processing the images in a slightly different way, using a single side-band, complex transfer function for CTF correction, it is possible to keep the Fourier components from the two diffracted beams computationally separate. It would thus be possible to completely correct for the curvature of the Ewald sphere and thus increase the “depth of field” at high resolution in all micrographs. The amount of defocus *ΔF*, needed to separate the two pseudo-Friedel-related Fourier components in the image is ΔF=Dd/2λ where *D* is the particle diameter, *d* is the resolution and *λ* is the wavelength. For 100 Å particles, a defocus of only ΔF=1 µm would fully separate the 4 Å Fourier components and allow complete correction for Ewald sphere curvature. For an image of a 1000 Å diameter virus, a defocus of 10 µm would be required to separate the 4 Å Fourier components, but less for higher spatial frequencies. Such an approach would also allow micrographs created with lower-energy electrons (e.g. 100 keV) to be used to obtain accurate structures without any deleterious signal loss due to Ewald sphere curvature. This approach is explored in detail in an accompanying paper [28].

## 5 Conclusions

The measurements reported here show a loss of contrast for single particles embedded in ice which is caused by the fluctuating charge present in and on the insulating layer of ice. Fortunately, the fluctuations are small and impose an envelope function that limits transfer of information from the specimen by only a few percent, even at atomic resolution and low-energy. This means that several potential advantages to working at lower energies and high defocus will not be limited by the effects of charge buildup on the specimen. Bearing in mind this and the measurements of the dynamics of charge buildup reported in the accompanying paper [13] we therefore conclude that for a single particle cryoEM specimen on a conductive support, the primary cause of information loss beyond that caused by radiation damage is not charge, but instead physical movement of the specimen during the beginning of irradiation.

## Appendix A Supplementary materials

Supplementary Data S1 Supplementary Raw Research Data. This is open data under the CC BY license http://creativecommons.org/licenses/by/4.0/ Supplementary Data S1

Supplementary Data S2 Supplementary Raw Research Data. This is open data under the CC BY license http://creativecommons.org/licenses/by/4.0/ Supplementary Data S2

Supplementary Data S3 Supplementary Raw Research Data. This is open data under the CC BY license http://creativecommons.org/licenses/by/4.0/ Supplementary Data S3

Supplementary Data S4 Supplementary Raw Research Data. This is open data under the CC BY license http://creativecommons.org/licenses/by/4.0/ Supplementary Data S4

Supplementary Data S5 Supplementary Raw Research Data. This is open data under the CC BY license http://creativecommons.org/licenses/by/4.0/ Supplementary Data S5

Supplementary Data S6 Supplementary Raw Research Data. This is open data under the CC BY license http://creativecommons.org/licenses/by/4.0/ Supplementary Data S6
