# Supplementary material for: Microscopic charge fluctuations cause minimal contrast loss in cryoEM
Source: Ultramicroscopy. 2018 Apr;187:56–63. doi: 10.1016/j.ultramic.2018.01.011 (PMC5862660; doi:10.1016/j.ultramic.2018.01.011)
Supplement: Supplementary Data S6 — Supplementary Raw Research Data. This is open data under the CC BY license http://creativecommons.org/licenses/by/4.0/ [file mmc6.pdf]

Supplementary Material

Supplementary Movie 1 The bee swarm effect on a holey carbon foil with plunge-frozen amorphous ice. The movie is the complete time series of images presented in the upper left panel of Fig. 2, where the field of view is cropped to (10 μm)^2 and the frames have been aligned and compressed to create the (1024 px)$^2\times$ 8 bit movie file.

Supplementary Movie 2 The bee swarm effect on a holey carbon foil with plunge-frozen amorphous ice. The movie is the complete time series of images presented in the middle left panel of Fig. 2, where the mean intensity is subtracted from each frame, the field of view is cropped to (10 μm)^2, and the frames have been compressed to create the (1024 px)$^2\times$ 8 bit movie file.

Supplementary Movie 3 The bee swarm effect on a holey gold foil with plunge-frozen amorphous ice. The movie is the complete time series of images presented in the upper right panel of Fig. 2, where the field of view is cropped to (10 μm)^2 and the frames have been aligned and compressed to create the (1024 px)$^2\times$ 8 bit movie file.

Supplementary Movie 4 The bee swarm effect on a holey gold foil with plunge-frozen amorphous ice. The movie is the complete time series of images presented in the middle right panel of Fig. 2, where the mean intensity is subtracted from each frame, the field of view is cropped to (10 μm)^2, and the frames have been compressed to create the (1024 px)$^2\times$ 8 bit movie file.

Supplementary Movie 5 Movie shows a series of micrographs taken at increasing amounts of defocus on the specimen shown in Fig. 4a. The defocus of the first 11 frames is nominally -0 to -10 μm in steps of -1 μm were each is offset from the nominal value by -100 nm. The subsequent frames 12 to 21 are the same as 1 to 10 in reverse order. The individual, Bragg diffracted sidebands are increasingly delocalised from the particles at increasing magnitudes of defocus. Defocus series like the one shown here were used for the data presented in Figs. 4–6. Each frame was binned 4$\times$ and cropped to create the 8 bit movie file.
